# Word Frequency and Content Analysis Approach to Identify Demand Patterns in a Virtual Community of Carriers of Hepatitis C

**DOI:** 10.2196/ijmr.2384

**Published:** 2013-07-04

**Authors:** Paulo Roberto Vasconcellos-Silva, Darlinton Carvalho, Carlos Lucena

**Affiliations:** ^1^Oswaldo Cruz InstituteNational School of Public HealthOswaldo Cruz FoundationRio de JaneiroBrazil; ^2^School of Medicine and SurgeryDEMESPFederal University of the State of Rio de Janeiro - UNIRIORio de JaneiroBrazil; ^3^Software Engineering LaboratoryDepartment of Computer SciencePontifical Catholic University of Rio de JaneiroRio de JaneiroBrazil

**Keywords:** Internet, online communities, hepatitis C virus carrier, social support, qualitative research, content analysis, social behavior

## Abstract

**Background:**

Orkut, a Brazilian virtual social network, is responsible for popularization of the Internet among people of low income and educational level. It’s observed that rapid growth of virtual communities can be reached by low cost Internet access in community local area network houses. Orkut poses an important social resource for Brazilian patients with chronic conditions like hepatitis C virus (HCV) carriers, who face several obstacles in adapting to everyday difficulties.

**Objective:**

Identify Patterns of Recurring Demands (PRD) expressed in messages posted by members of virtual communities dedicated to HCV carriers.

**Methods:**

Pre-selection: we identified terms commonly associated to HCV on generic Internet searches (primary Keywords - Kps); Kps were used to identify the most representative HCV communities in a virtual community site (Orkut); all messages published along 8 years on all topics of the community were collected and tabulated; the word frequency was used to construct a “word cloud” (graphic representation of the word frequency) on which was applied a content analysis technique.

**Results:**

The most cited terms expressed: search for information about medications (prescribed and “forbidden”); emphasis on counting time, which were interpreted as surviving expectations; frequent mention of God, doctors, and “husbands” (female carriers were 68%). These elements provided material for further research – they will be useful in the construction of categories in discourse analysis.

**Conclusions:**

The present work is a disclosure of preliminary findings considered original and promising. The word frequency/content analysis approach expressed needs of social support and material assistance that may provide subsidies for further qualitative approach and public health policies aimed to HCV carriers. The study of PRD by word frequency may be useful in identifying demands underestimated by other means.

## Introduction

Psychological complications and physical symptoms arising from hepatitis C are well-known and described as consequence and conditioning factor for recurrence [1]. Therefore, this combination of problems, treatment side effects, perspective of recurrence, and need for radical lifestyle changes, brings challenges to hepatitis C virus (HCV) carriers. It would be impossible to tolerate such obstacles for a long time without the social support from spouses, relatives, friends, and other HCV carriers. It is believed that the support coming from specialized virtual communities (VCs) represents an important resource for HCV patients who encounter obstacles in adapting to everyday difficulties. The Internet offers several tools for organization of virtual networks of chronic patients, which are here presented as an object of study.

The purpose of the present research note is to identify patterns of recurring demands (PRD) posted by members of VCs organized by Brazilian HCV carriers. These preliminary results raised promising hypothesis that will be used by qualitative research experts. A broader "discourse analysis" will be organized based on VC postings and focus groups with HCV carriers in assistance environments.

## Methods

**Figure 1:**
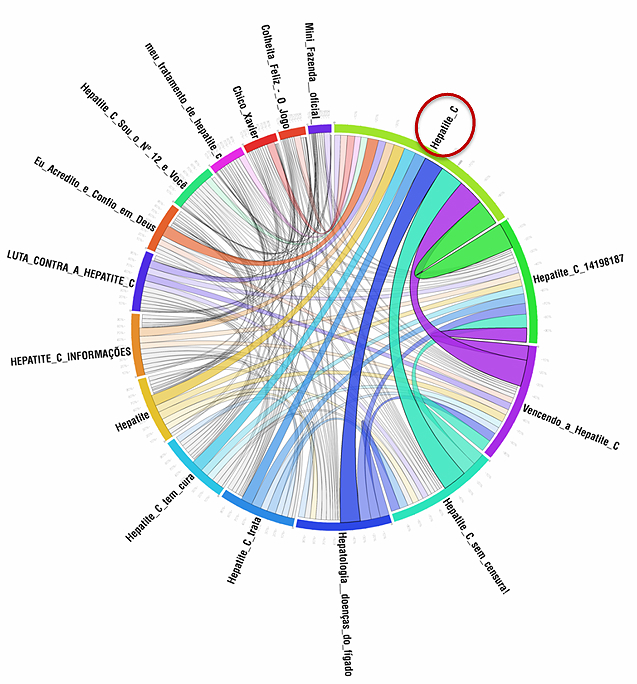
The Community Association Map (CAM) shows interrelationship between communities of users with the Dominant Community and confirm their dominance around a core of common interests.

**Figure 2:**
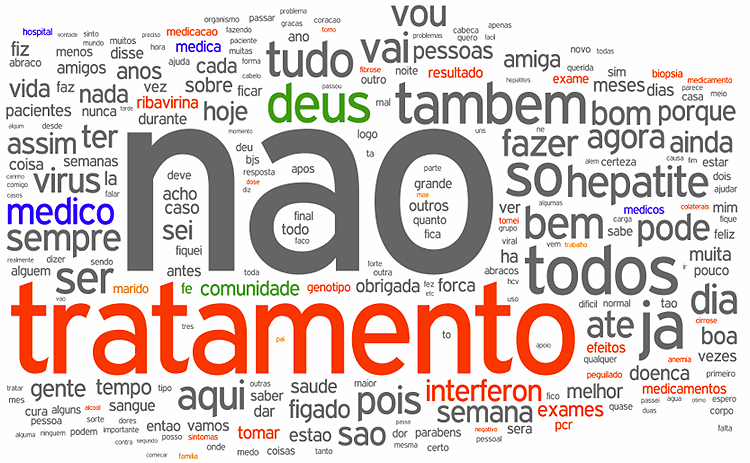
Word cloud of the Dominant Community forum.

Methodology can be summarized as an incremental Internet search in decreasing dimensions of coverage, as described in detail by Carvalho et al [2]. We employed open access algorithms (Google insights) to identify the most common terms associated to HCV in general searches—referred here as primary Keywords (Kps) as expressions of casual and indistinct interest. Orkut was chosen because of its long existence (established in January 2004) and its popularity in Brazil. In Orkut, thematic discussions are organized into “topics” in which messages are posted. The site also has special features for searching in which Kps were applied to disclose “specialized” (carriers) VCs. The VCs that mentioned any of the Kps at least once were selected. We assessed the "relevance weight" among Orkut HCV communities by choosing the ones in which Kps were more frequent. Associations of Kps applied among the most popular and active VCs can neutralize bias caused by arbitrary choices in the pre-selection process. This criteria is based on other algorithms like “page rank” [3], which estimates the relevance of a site using the number of highest expression links directed to it. From this set of VCs, the most representative were chosen by its time of existence, number of members, and mainly by the Kps frequency in discussions. This Dominant Community was considered for study. The relationship of the Dominant Community with its peers was studied through the Community Association Map (CAM, Figure 1), which defines the interrelationships between communities [4] to portray and confirm their dominance around a core of common interests. We developed scripts to collect and tabulate all messages published on all topics over eight years of the community’s existence. A “word cloud” (in which word’s size is proportional to it’s frequency) was generated in wordle to provide a graphic representation of the word frequency (Figure 2).

## Results

### Hepatitis C

The main *Kps* associated with “*Hepatite C*” found on Google were (in Portuguese): *consulta hepatite c; cura hepatite c; exame hepatite c; hepatite c cronica; hcv; medicamento hepatite c; medico hepatite c; prevencao hepatite c; remedio hepatite c; sintomas hepatite c; transmissao hepatite c; tratamento hepatite c; vacina hepatite c; virus hepatite c.* The search using these *Kps* in Orkut resulted in 1476 communities. The highest occurrences of *Kps* were found in: *Hepatite C* = 588 topics, *HIV - BR* = 208; *hepatite C* = 183; *Hepatologia: doenças do fígado* (Hepatology: liver disease) = 107; *hepatite c informações* = 129. In these communities, 20,000 messages were posted by 9066 members (may be overestimated - users might belong to multiple communities).

“Hepatite C” (ID 216788 on Orkut) was considered the *Dominant Community* because it gathered the largest volume of public content. It was created by a HCV carrier a few months after the Orkut’s launch. It is not associated to any commercial or government institution, and in 2453 days, it gathered 1292 members—68% women and 32% men.

### Content of Messages Posted on “Hepatite C”

**Figure 3:**
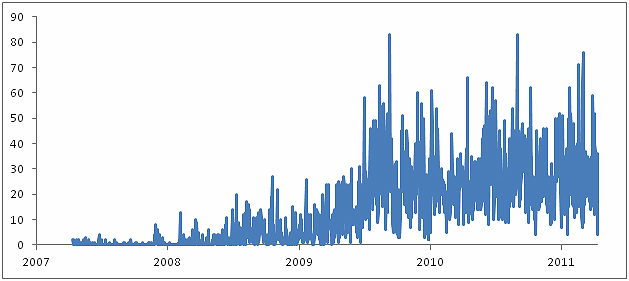
Historical evolution of messages per day posted at the Dominant Community forum.

The message volume in the Dominant Community remained low until the first quarter of 2009, thereafter the number of messages increased significantly, as shown in Figure 3, which matches with the popularization of the site in Brazil [5]. Of the total 47,005 terms found on all topics in 8 years of available content, the most recurring words in frequency analysis (discarding articles, prepositions, and numerals) are presented in Table 1, which also presents a subset of other prominent words (that refers to HCV therapy and potentially toxic drugs to carriers). From the most cited drugs, *interferon* and *ribavirin* shared six of the eight stronger relationships between drugs cited in the same message. These findings suggest a demand by the strong association between ribavirin and interferon—its continuing use is a frequent problem to patients of Brazilian healthcare system [6]. The three types of information that are most frequently found with this association are *preço* (price), *bula* (medication user instructions), and *efeitos colaterais* (side effects).

**Table 1:** Subset of prominent words from the *Dominant Community* forum (translated to English).

Words		Citations
**Most recurring words in frequency analysis**		
	Treatment	9581
	God	4077
	Hepatitis	3329
	Physician	2800
	Virus	2674
	Interferon	2281
	Husband	971
	Well	3411
	Do	2822
	Ribavirin	1288
	Patients	1279
**Recurrent words related to the passage of time**		
	Day	3156
	Always	2852
	Week	2285
	Then	2170
	Years	1771
	Months	1454
	During	1199
**Therapies for the control of HCV** ^a^		
	Interferon	1554
	Ribavirin	1048
	Erythropoietin	317
	PEGASYS	201
	Folic acid	157
	PEGINTRON	130
	Filgrastim	109
	Silymarin (alternative treatment)	63
**Potentially toxic drugs to CHCV** ^b^		
	TYLENOL	121
	Omeprazole	80

^a^HCV=hepatitis C virus

^b^CHCV=chronic hepatitis C virus

## Discussion

### Principal Findings

The PRD on the Internet are presented here as a low cost method easily applicable for guiding qualitative researchers on data collection. Hypotheses, linking concepts, and “bounding ideas” are essential for the portrayal of social support and can be easily weakened by bias and personal assumptions - which can be preventable by the PRD analysis. The HCV carriers are vulnerable to several psychological conditions and depressive symptoms that are usually identified and reported among them [7,8]. The Identification of PRD in communities with chronic diseases may expand our comprehension about their needs for social networks, presenting demands perhaps underestimated by public health policy makers. It’s interesting to notice that the results presented here support other conclusions recently reached by other methods. Sousa [6] describes carriers expressing their suffering strongly attached to expectations of obtaining medicines and healing in the context of the passage of the weeks, months, and years of survival. The elements described herein provide a basis for further, more detailed, research, in which the PRD are consolidated into central ideas for the construction of analysis categories. The primary purpose of the paper was to furnish unbiased material to a qualitative approach, which could reach findings applicable beyond the immediate boundaries of the study. According to qualitative research literature [9,10], it’s especially effective in cultural research, which deals with values, opinions, and perspectives that can be generalized in a broader view.

Another interesting aspect concerning the method refers to the study of VC through algorithms, a field underused so far despite several remarkable alternatives. In addition to reduced costs compared to conventional field research, there is an opportunity to capture discourses posted in moments of desperate need for support. Here, Internet VCs seem to transcend their merely informative context [11], and acquire a unifying force aimed at overcoming great obstacles [12]. Besides posting messages on topics for mutual enlightenment and social support, maybe HCV carriers feel more comfortable talking about personal difficulties and living conditions when compared to conventional medical consultation enviroment. In general, stigmatized diseases or health conditions encourage individuals to use Internet as a main source of information and environment for sharing experiences [12-14]. Such preferences are not limited to the possibility of hiding identities in the face of uncomfortable topics, but also include the VC role of social support. Pattern analysis identified frequent use of words that suggest a need for spiritual support (God: 4077 citations) and social support (husband, 971). Words indicating the need for material support/care were extensively mentioned: *treatment* (9581), *doctor* (2800), and *interferon* (2281), and the association between *Interferon* and *Ribavirin* (also described by Sousa [6]). These results reinforce evidence that patients with chronic diseases have a distinct profile of engagement in virtual communities. We found agile dissemination of certain content and thematic consistency associated with interest in news about innovative therapies (new formulations of interferon; alternative therapies).

### Conclusion

The present work is a disclosure of preliminary findings considered original and promising. The word frequency / content analysis approach expressed needs of social support and material assistance that may provide subsidies for further qualitative approach and public health policies aimed to HCV carriers.

Research on PRD requires small resources in its development in contrast with important outcomes in terms of depiction of demands from patients with chronic diseases underestimated by other perspectives. The word frequency and content analysis can furnish hypotheses, linking concepts, and “bounding ideas,” which are essential for the portrayal of collective ideas and social support demands. The present findings describe some evidence of need for social and material support that may subside public policies aimed at carriers of HCV.

## Abbreviations

CAM: Community Association Map
HCV: hepatitis C virus
Kps: primary keywords
PRD: patterns of recurring demands
VC: virtual community
